# Supplementary material for: A conserved lysine/arginine-rich motif is essential for the autophagic degradation of potyviral 6K1 protein and virus infection
Source: J Virol. 2025 Feb 10;99(3):e02183-24. doi: 10.1128/jvi.02183-24 (PMC11915830; doi:10.1128/jvi.02183-24)
Supplement: Supplemental material — Table S1, Figures S1 to S3, and Data S1. [file jvi.02183-24-s0001.pdf]

Supplementary Information

A conserved lysine/arginine-rich motif is essential for the autophagic degradation of potyviral 6K1 protein and virus infection

Weiyao Hu,[a] Changhui Deng,[a] Li Qin,[a] Peilan Liu,[a] Linxi Wang,[a] Xiaoqin Wang,[a] Wei Shi,[a] Asma Aziz,[a] Fangfang Li,[b] Xiaofei Cheng,[c] Aiming Wang,[d] Zhaoji Dai,[a#] Xiaohua Xiang,[e#] Hongguang Cui[a#]

[a]Key Laboratory of Green Prevention and Control of Tropical Plant Diseases and Pests (Ministry of Education) and School of Tropical Agriculture and Forestry, Hainan University, Haikou, China; [b]State Key Laboratory for Biology of Plant Diseases and Insect Pests, Institute of Plant Protection, Chinese Academy of Agricultural Sciences, Beijing, China; [c]College of Plant Protection/Key Laboratory of Germplasm Enhancement, Physiology and Ecology of Food Crops in Cold Region of Chinese Education Ministry, Northeast Agricultural University, Harbin, China; [d]London Research and Development Centre, Agriculture and Agri-Food Canada, London, Ontario, Canada; [e]Haikou Cigar Research Institute, Hainan Provincial Branch of China National Tobacco Corporation, Haikou, China.

#Correspondence: zhaoji.dai@hainanu.edu.cn; xiangxiaohuacaas@163.com; hongguang.cui@hainanu.edu.cn.

Weiyao Hu, Changhui Deng, and Li Qin contributed equally to this work. Position was determined by ascending chronological order based on the time in which these authors started with the project.

Supplemental Table S1

Supplementary Table S1 Primers used in this study

Primer Name	Sequence (5'→3')	Use
P-GFP-F	CTTTGTTCTGCACCAAGCAGGAATGAGTAAAGGAGAAGAACTTT	
P-GFP-R	GACAGTCTCACCAGCCTGATGAAGGACGAAATCTTTGTATAGTTCATCCATGCC	
C2-F	TTTCGTCCTTCATCAGGCTGGTGAGACTGTCGATGC	Plasmid Construction: pHNu-GFP
C1-R	TTTACTCATTCCTGCTTGGTGCAGAACAAAGTCATCA	
C-F	ATATAGACGTCATATGTGATTTGCATG	
C-R	ATATAGTCGACTTTTTTTTTTTTTTTTTTTTAGCGCCAATTATGAAACCGC	
PV-C-inf-F	TGAATTGGCAGTCGAGGACGTC	
PV-C-inf-R	GCCAAATGTTTGAACGATCG	Plasmid construction: pHNu-NIb^{GFP}
NIb-GFP-R	TTCTCCTTTACTCATGTGCAGAACAAAGTCATCACC	
NIb-GFP-F	GACTTTGTTCTGCACATGAGTAAAGGAGAAGAACTTTTC	
PV-Stu-inf-F	GGTTCTATTGCTTGAGCCAAC	
CIMyc-R	GTTTCCATCAGATCTTCTTCAGAGATCAGTTTCTGTTCATGCATGACAGTTTCCATAG	Plasmid construction: pHNu-GFP-CI^{Myc}
CIMyc-F	CTGATCTCTGAAGAAGATCTGATGGAAACAGTGATGCACCAGAGTACAGAAGAGATTGG	
PV-Aat-inf-R	TCACAGCATGCAAATCACATATG	
6K1^{Myc}-F	ATCTCTGAAGAAGATCTGGCAGTTAATCATCAGAGCAT	
6K1^{Myc}-R	ATGATTAACTGCCAGATCTTCTTCAGAGATCAGTTTCTGTTCATGATTAACTGCGTCATTATC	Plasmid Construction: pHNu-GFP-6K1^{Myc}
2280-F	TGTTTCCATCGACACGCTCT	
4070-R	ACACGGATTTATTCCTCATC	
^{Myc}P3-F	GAACAGAAACTGATCTCTGAAGAAGATCTGGGCAGTGACCCACGAACTCT	Plasmid Construction: pHNu-GFP- ^{Myc}P3
^{Myc}P3-R	CAGATCTTCTTCAGAGATCAGTTTCTGTTCGCTACCTCCAACACGGTACATTTTCATC	
Q-H-F	TGATGTGAAGCATCACGCGAGCAAGAGACC	
Q-H-R	GGTCTCTTGCTCGCGTGATGCTTCACATCAT	
A-K-F	GATGTGAAGCATCAAAAGAGCAAGAGACCAGC	Plasmid Construction: pHNu-GFP-^{Myc}P3(Q-H), pHNu-GFP-^{Myc}P3(A-K), pHNu-GFP-^{Myc}P3(QA-AQ), pHNu-GFP-^{Myc}P3(Q-A), pHNu-GFP-^{Myc}P3(A-Q)
A-K-R	CTGGTCTCTTGCTCTTTTGATGCTTCACATC	
QA-AQ-F	TGATGTGAAGCATGCACAGAGCAAGAGACCAGCCGAAG	
QA-AQ-R	GGTCTCTTGCTCTGTGCATGCTTCACATCATTGTTC	
QA-F	AATGATGTGAAGCATGCAGCGAGCAAGAGAC	
QA-R	TCTCTTGCTCGCTGCATGCTTCACATCATTG	
AQ-F	TGTGAAGCATCAACAGAGCAAGAGACCAGC	
AQ-R	TGGTCTCTTGCTCTGTTGATGCTTCACATC	
K3A-F	GCATCAAGCGAGCGCTAGACCAGCCGAAGCTAAGCT	
K3A-R	GCTTCGGCTGGTCTAGCGCTCGCTTGATGCTTCACAT	
E11A-F	CGAAGCTAAGCTTGCCCAGATCGTAGCTTTTGTCGC	
E11A-R	AAAGCTACGATCTGGGCAAGCTTAGCTTCGGCTGGT	
A15R-F	TGAACAGATCGTACGATTTGTCGCACTTATGATGATG	
A15R-R	ATAAGTGCGACAAATCGTACGATCTGTTCAAGCTTAG	
L19A-F	GTAGCTTTTGTCGCAGCAATGATGATGGTTTTTGATACTG	
L19A-R	AACCATCATCATTGCTGCGACAAAAGCTACGATCT	
M22A-F	GCACTTATGATGGCAGTTTTTGATACTGATAGAAGC	
M22A-R	ATCAGTATCAAAAACTGCCATCATAAGTGCGACAAAAG	To mutate 17 conserved residues of 6K1
D25A-F	TGATGATGGTTTTTGCAACTGATAGAAGCGACTGTGT	
D25A-R	CGCTTCTATCAGTTGCAAAAACCATCATCATAAGTG	
D27A-F	TGGTTTTTGATACTGCAAGAAGCGACTGTGTCTACAA	
D27A-R	CACAGTCGCTTCTTGCAGTATCAAAAACCATCATCA	
R28A-F	GTTTTTGATACTGATGCTAGCGACTGTGTCTACAAAGT	
R28A-R	GACACAGTCGCTAGCATCAGTATCAAAAACCATCATC	
S29A-F	TTGATACTGATAGAGCAGACTGTGTCTACAAAGTGCT	
S29A-R	GTAGACACAGTCTGCTCTATCAGTATCAAAAACCATC	
D30A-F	ATACTGATAGAAGCGCTTGTGTCTACAAAGTGCTCAA	
D30A-R	CTTTGTAGACACAAGCGCTTCTATCAGTATCAAAAAC	

V32A-F	TAGAAGCGACTGTGCATACAAAGTGCTCAATAAGCTC	
V32A-R	TTGAGCACTTTGTATGCACAGTCGCTTCTATCAGTA	
K34A-F	CGACTGTGTCTACGCTGTGCTCAATAAGCTCAAGAAT	
K34A-R	AGCTTATTGAGCACAGCGTAGACACAGTCGCTTCTAT	
L36A-F	TGTCTACAAAGTGGCAAATAAGCTCAAGAATGTCATG	
L36A-R	TTCTTGAGCTTATTTGCCACTTTGTAGACACAGTCGC	
K38A-F	CAAAGTGCTCAATGCACTCAAGAATGTCATGGGAGT	
K38A-R	ATGACATTCTTGAGTGCATTGAGCACTTTGTAGACAC	
L39A-F	AAGTGCTCAATAAGGCAAAGAATGTCATGGGAGTTGT	
L39A-R	CCATGACATTCTTTGCCTTATTGAGCACTTTGTAGA	
K40A-F	GCTCAATAAGCTCGCTAATGTCATGGGAGTTGTTGAT	
K40A-R	ACTCCCATGACATTAGCGAGCTTATTGAGCACTTTGT	
V51A-F	ATAATGACGCAGCAAATCATCAGAGCAT	
V51A-R	GCTCTGATGATTTGCTGCGTCATTATC	
NIb-6K1-F	TTTGTTCTGCACCAAGCGAGCAAGAGACCAGCCGAAG	Plasmid construction: pHNu//6K1 and pHNu//6K1^{GFP}
NIb-6K1-R	CTGGTCTCTTGCTCGCTTGGTGCAGAACAAAGTCATC	
6K1-CP-F	TTAATCATGATTTCGTCCTCCATCAGGCTGGTGAGACTGTCGATGCAG	
6K1-CP-R	CAGCCTGATGGAGGACGAAATCATGATTAACTGCGTCATTATC	
6K1-GFP-F	GACGCAGTTAATGCAGCTAGTAAAGGAGAAGAACTTTTC	
6K1-GFP-R	CTTCTCCTTTACTAGCTGCATTAACTGCGTCATTATCAAC	
mCherry-F	ATATATCTAGAATGGTGAGCAAGGGCGAGGAG	Plasmid construction: pCaM-mCherry
mCherry-R	ATATAGGTACCTTAGGATCCCTTGTACAGCTCGTCCATGC	
6K2-Xho-F	CTACAAATCTATCTCTCTCGAGATGAGTACAGAAGAGATTGGG	Plasmid construction: pCaM-6K2-mCherry
6K2-Xba-R	GCCCTTGCTCACCATTCTAGAGTGTGACACGTCATCTCC	
NbATG8a-Bam-F	GACGAGCTGTACAAGGGATCCATGGCCAAAAGCTCCTTCAAATTG	Plasmid construction: pCaM-mCherry-NbATG8a
NbATG8a-Kpn-R	GGGAAATTCGAGCTCGGTACCTTAGAACGATCCGAATGTATTC	
NbATG8f-Bam-F	GACGAGCTGTACAAGGGATCCATGGCAAAGAGTTCATTCAAGC	Plasmid construction: pCaM-mCherry-NbATG8f
NbATG8f-Kpn-R	GGGAAATTCGAGCTCGGTACCTTACACCAAGTTAAAGTCCCCAAATG	
GFP-F	ATATATCTAGAATGAGTAAAGGAGAAGAACT	Plasmid construction: pCaM-GFP
GFP-R	ATATAGGTACCTTAGGATCCTTTGTATAGTTCATCCATGCC	
6K1-Xho-F	CTACAAATCTATCTCTCTCGAGATGGCGAGCAAGAGACCAGCCG	Plasmid construction: pCaM-6K1-GFP
6K1-Xba-R	CTTCTCCTTTACTCATTCTAGAATGATTAACTGCGTCATTATC	
6K1Myc-Xba-inf-F	CGAGCTTTCGCGAGCTCGACTCTAGAATGGCGAGCAAGAGACCAGCCGAAG	Plasmid construction: pCaM-6K1^{Myc}
6K1Myc-Kpn-inf-R	GGGAAATTCGAGCTCGGTACCTTACAGATCTTCTTCAGAGATCAGTT	
NIb-XhoI-inf-F	TCTCTACAAATCTATCTCTCTCGAGGGAGAAGAAAAATGGTTGTATGCAC	pCaM-NIb^{Myc}
NIbMyc-XbaI-inf-R	GCTCGGTACCTTAGGATCCTCTAGACAGATCTTCTTCAGAGATCAGTTTCTGTTCTTGGTGCAGAACAAAGTCATCACCT	
NbNBR1-Xho-inf-F	CTCTCTACAAATCTATCTCTCTCGAGATGGCCATGGAGTCTGCTATTGTG	pCaM-NBR1^{Myc}
NbNBR1Myc-Xba-inf-R	GCTCGGTACCTTAGGATCCTCTAGACAGATCTTCTTCAGAGATCAGTTTCTGTTCCTGCTCTCCAGCAATAAGAT	
dsNbATG7-Sac-F	ATATAGAGCTCCAAGGTTGGCTGTATCAGCTGCTG	Plasmid construction: p2300s-intron-dsATG7
dsNbATG7-Bam-R	ATATAGGATCCCAAGACCTTCTGCTTCCACTGCTGG	
dsNbATG7-Pst-F	ATATACTGCAGCAAGGTTGGCTGTATCAGCTGCTG	
dsNbATG7-Xba-R	ATATATCTAGACAAGACCTTCTGCTTCCACTGCTGG	
dsGUS-Sac-F	ATATAGAGCTCTACGTCCTGTAGAAACCCCAACC	Plasmid construction: p2300s-intron-dsGUS
dsGUS-Bam-R	ATATAGGATCCATCACTTCCTGATTATTGACCC	
dsGUS-Pst-F	ATATACTGCAGTACGTCCTGTAGAAACCCCAACC	
dsGUS-Xba-R	ATATATCTAGAATCACTTCCTGATTATTGACCC	
PVMV-CP-F	TACACGGGCATTGCAGTCAC	RT-qPCR
PVMV-CP-R	TGCTCTTCGCCATCAACCAT	
CcActin-q-F	TCAGCCACACAGTTCCCATC	
CcActin-q-R	TAACAATTTCCCGCTCGGCT	
NbActin-q-F	AAAGACCAGCTCATCCGTGGAGAA	
NbActin-q-R	TGTGGTTTCATGAATGCCAGCAGC	
NbATG7-q-F	GTTGGCCGCTTCGCAATTAT	
NbATG7-q-R	TTTAGTTCCCATCCCACGGC	
GFP-q-F*	CCGACAAGCAAAAGAACGGC	

GFP-q-R*	AGGGCAGATTGTGTGGACAG
NBR1-q-F	TAATCACAGTGATGGGACTG
NBR1-q-R	GTAATCAGCATCATTTCCCA
NIb-q-F	ACACAATAGCACCCTGGAGT
NIb-q-R	CCATCAGCTGTAAGTATAGGT

Note: The primers indicated with asterisks were designed in a previous publication (Hu et al., 2023).

Supplementary Figures

Supplemental Figure Legends:

Supplemental Figure S1 Infectivity test of mutated PVMV clones in *N. benthamiana*. The GFP signals were examined under a handheld UV lamp in a darkness room at 10 dpi.

Supplemental Figure S2 Infectivity test of mutated PVMV clones in *C. chinense*. The GFP signals were examined under a handheld UV lamp in a darkness room at 15 dpi.

Supplemental Figure S3 Infectivity test of pHNu//6K1 variants in *N.benthaminana* and *C. chinense*. (A) Infectivity test of pHNu//6K1 variants in *N. benthamiana*. The representative plants were photographed at 5 dpi. (B) Symptom phenotypes triggered by pHNu//6K1 variants in *N. benthamiana* at 10 dpi. (C, E) RT-qPCR analysis of viral accumulation levels. The upper non-inoculated leaves of *N. benthamiana* 10 dpi (C) and *C. chinense* at 15 dpi (E), were sampled for the assays. The expression levels of *NbActin* and *CcActin* transcripts were determined to normalize the data. Error bars denote standard errors from three biological replicates. Statistically significant differences, determined by an unpaired two-tailed Student's *t* test, are indicated by asterisks: *, $0.01<P<0.05$; NS, no significant difference. (D) Symptom phenotypes triggered by pHNu//6K1 variants in *C. chinense* at 15 dpi.

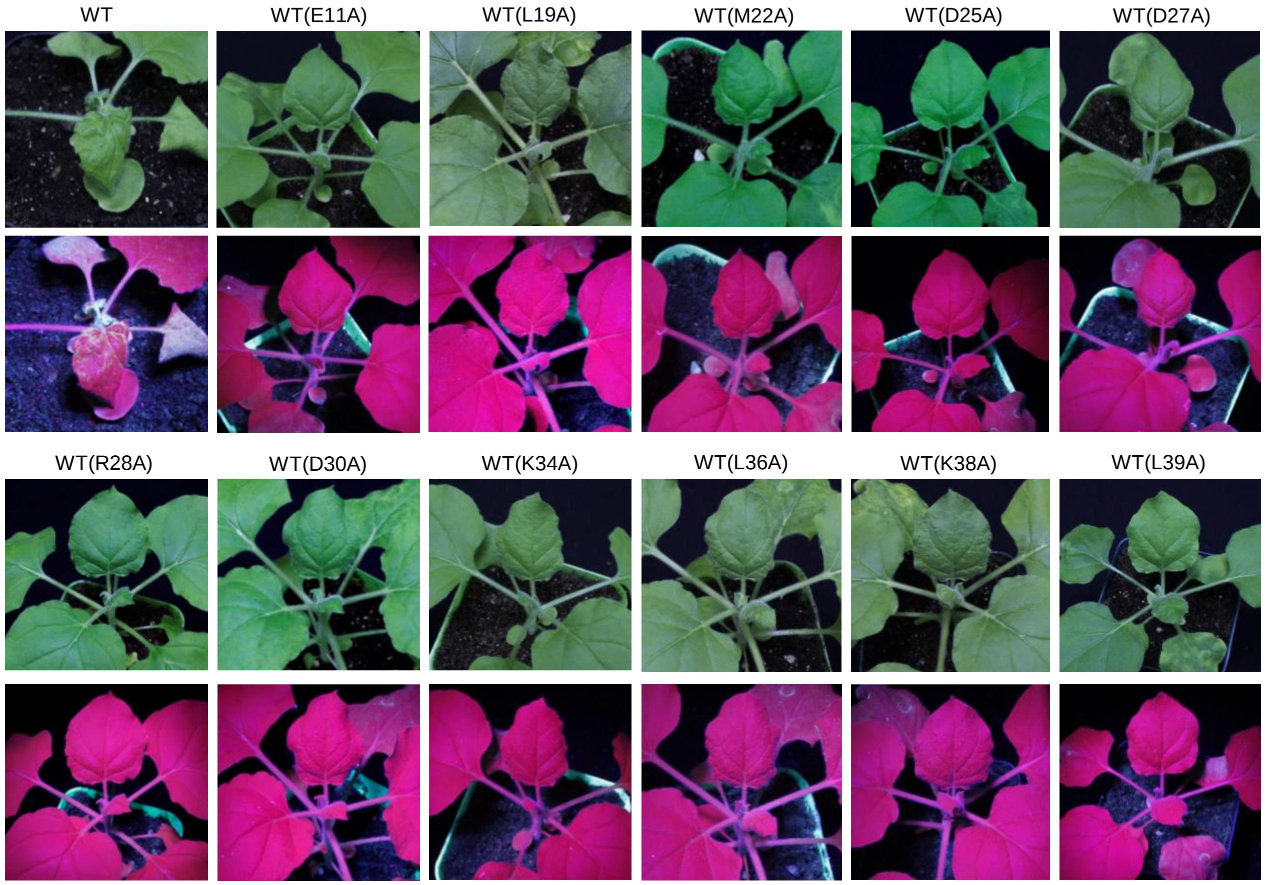

Supplemental Figure S1

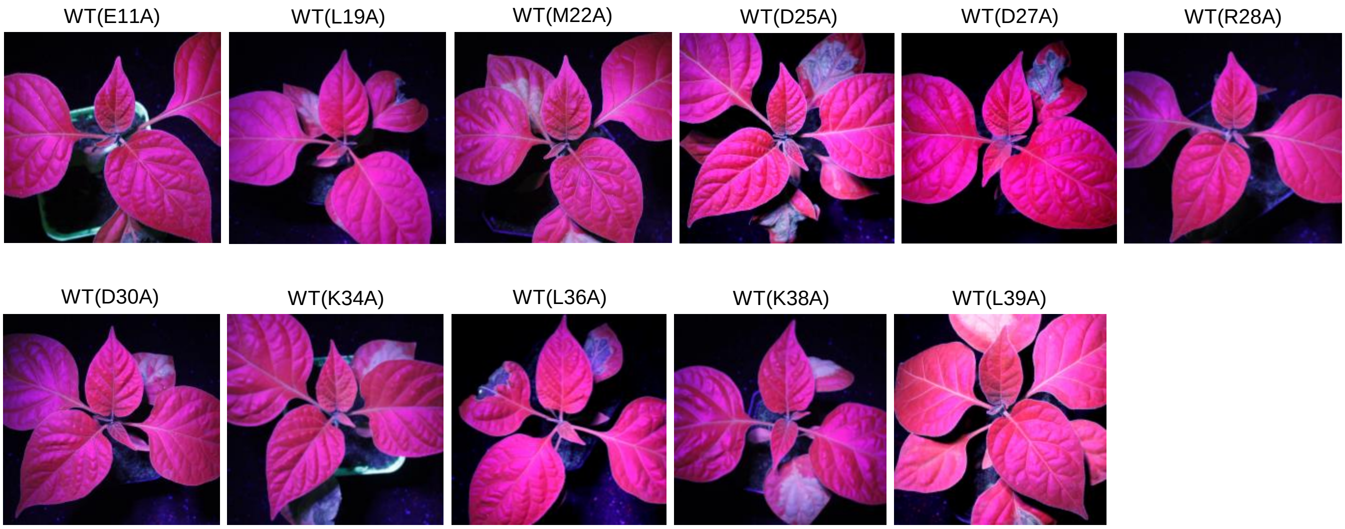

Supplemental Figure S2

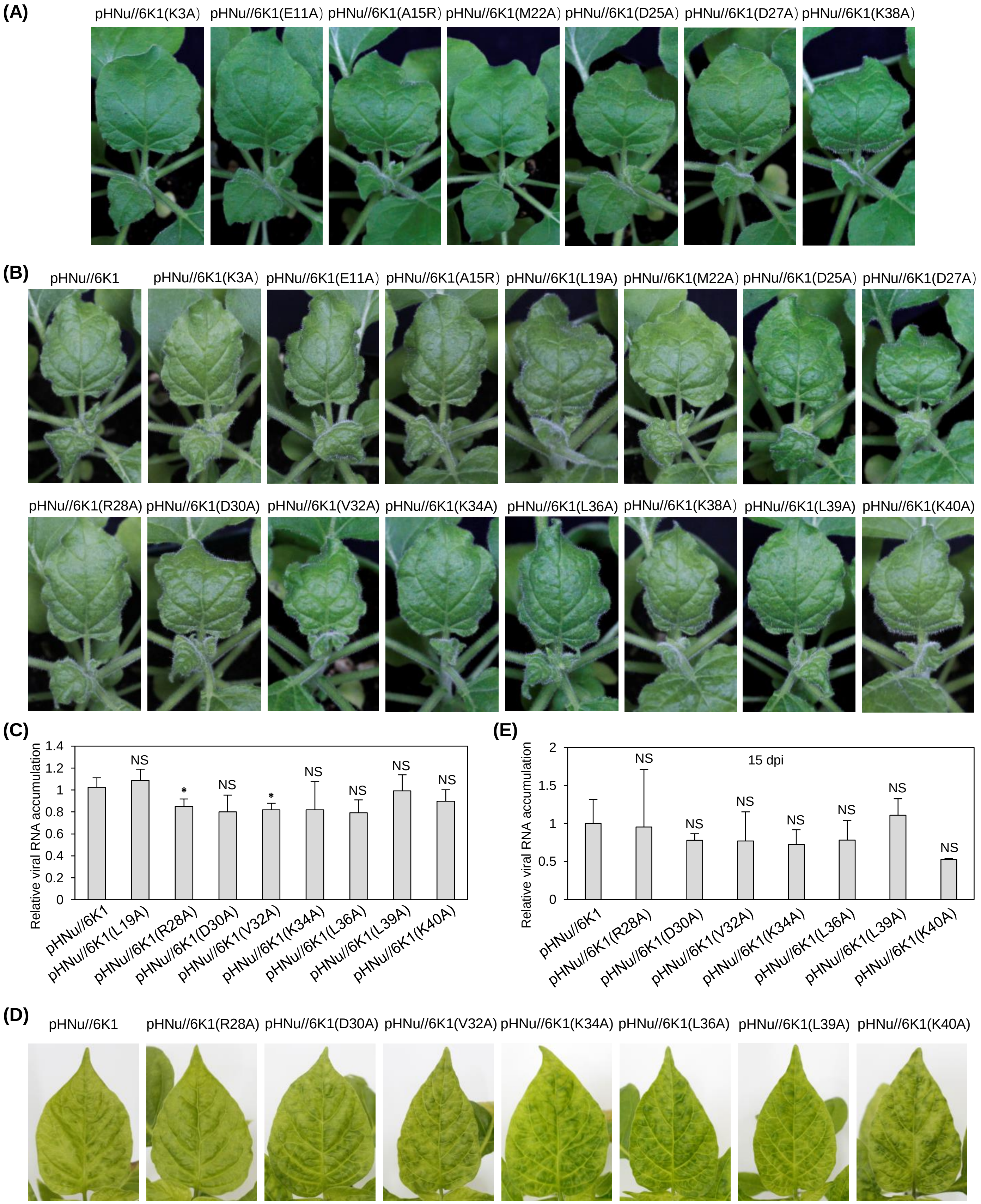

Supplemental Figure S3

Supplemental Data S1

Supplemental Data S1 A total of 115 sequences representing different potyviral 6K1s used for multiple alignment

>PVMV MN082715
ASKRPAEAKLEQIVAFVALMMMVFDTDRSDCVYKVLNKLKNVMGVVDNDAVNHQ
>AWMV EU410442
AKTVNEKNMERILAFLTLIMMMVDADKSDCVYKLLNKFKSVVGTIEQDVYHQ
>ApVY HM363516
AKRVSEARLEQAMAFVALILMAIDSERSDCVHKVLNKLKSLMSIADADVYHQ
>AV1 KJ830760
HKSKSQDTLQKIVAFVALIMMTFDADRSDCVYKILNKLKGIIGTIEYEVQHQ
>BBrMV DQ851496
SKTVFESGMERVVAVFALLAMIFDTSKSDAVFRILQKFKTCIASINNRVGFQ
>BaRMV DQ821938
SKNPFERNLEKVVAMMALFAMVFGSDKSSAVFNVLRNIKTVFGTLEDGVRYQ
>BCMV AY112735
AKNATQLQFEKIVAFMALLTMVIDTERSDAIFKILSKLKTVFQTMGENVQIQ
>BlCMV AJ312437
AKNATQLQFEKIVAFMALLTMLIDTERSDAIFKILSKLKTVFNTMGETVQIQ
>BYMV   MN509831
AKPNDMVALEKIVAVAASVLMIFDAERSDCVYKVLNKLKGILSTTTQDAYRFQ
>BtMV AY206394
ANKVYEKKLEKAVALMALFTMIFDTEKSGAVFSILRNIKSVFSTLGEEVKYQ
>BiMV KF649336
QSTTGVKNLEQVVAFITLVMMVFDSERSDCVFKTLNKLKGVVSTLDYGVRHQ
>BSVA JQ807999
AKTAVQLQFEKIIAVLALLTMCIDAERSDAIFRILSKIKTVFTTMGEDVKLQ
>BruMV JX867236
KSFDELKKLEHIVAFISLVVMVFDAERSDCVFKTLNKFRGIVGSLNSNVHHQ
>CalMoV KX013584
AKRESEAKLEQVVAFTALLLMLVDSERSDGVYKILSKLKGIMSSVDGPVYHQ
>CaYSV GQ421689
KKGSTELQLEQAIAFATLLTMLFDADRSDAVFRILQKIRSCTQIIGTTVEHQ
>ChiVMV AJ237843
ASKRPSEAKLEQIVAFVALMMMVFDTDRSDCVYKVLNKLRNVMGVVDNDAVNHQ
>ClYVV AB011819
SKSNDMVALEKVVAFTALVMMIFDGERSDCVYKILNKLKGIISTTTQDGYKFQ
>CSV AF499738
AKTHAEAKLEQIMARMALAAMMFDAQRSDAVFKVLSKIKTVLTSAGQSVHHQ
>CoSMV MK286375
YSNDTTTKKIEQIIAFSTLLLMLFDQERSDALFRTLMKCKGVFSGLGQMVQQQ
>CABMV AF348210
AKTHSQVHLEKIVAFMALLTMCVDAERSDAIFKILNKLKSVFGTMAEEVRVQ
>CVBV KY657266
KGPTEQNLEKVIAFMVLLAMFVDANRSDAIASVLNKVKNITTTLSTPVHHQ
>CeMV HQ676607
AKRVTEARLEQAMAFVALVLMAIDSDRSDCVYKVLNKLKSLMTIADADVYHQ
>CEVA JQ723475
HKRKSENDLERIVAVIAMIMMVFDSSRSDAVFKILNKLKAVFGTFNERVQFQ
>CRSV JN008909
AKRESEVRLEQIVAFMALVMMIFDNDRSDCVYRVLNKFKNIVSTAEQDVVHQ
>DapMV DQ299908
SKSAESQQYEKSLAFVALLLMVFDSERSDCVYKVLCKLRALTNICGERVQYQ
>DVY KU556609
AKNVENQKFEKIIAFIALVMMIFDTQRSDCVYKALTKIRSLTTICGETVKYQ
>DsMV AJ298033
AKTALQLQFEKIIAFLAIVTMCVDAERSDAVFRLLSKLKTVFATVGEDVRIQ

>VanMV KX505964
AKTALQLQFEKIIAFLAIVTMCIDSERSDAVFKLLSKLKTVFATVGENVRIQ
>DeCMV MK241979
AKSPYEQGLEKTVGIFALVAMIFDTARSDAVFRILSKLKTVFSLIDDKVHHQ
>DMV MH206616
ASNRTEQQRLEQIVAFVALVMMMFDANRSDCVYRILNKLKTLVSGAEGLVIHQ
>DOVA JX156422
AKSKDMFKLEQCVAFVALVLMLFDNERSDAVFKILNKLKTTFTTICGGVTHQ
>EAPV AB246773
AKTPVQCQFEKIVAFMALLTMCIDTERSDAIFKILNKLKVVFSTMGEDVKIQ
>FreMV FM206346
GKSPTEKSLERTVAMVALLAMVFDTERSDAVFKILSKIKSVFSTLGDEVKYQ
>FVY AM039800
SKEAIQLQFEKIIAFMALLTMCIDTERSDAIFKILSKLKTVFNTMGEDVMIQ
>GoMV LC228573
NKTYIERNFEKIIAFMALLTMMFDEKKSDAVFKCLGKIKTVFGTMDDVVRLQ
>HaMV AB818538
ANKRESETRLEQIVAFIALVLMVFDNERSDCVYRVMNKLKNVMSVAEQDVNHQ
>HarMV HQ161081
AKTAVQLQFEKIIAILALATMCIDAERSDAVFRILSKIKMAFSTVGEDVRLQ
>HiMV JQ395040
SKSKQEHIFEQIIGFLLLATLMFSPARSDVVFKVLNKVKSILTSTAPDCRFQ
>HyaMV KY828925
AKKECEVELERVIAFMALVMMVFDNERSDYVHRALSKVKSLMSSIDSDVKHQ
>IFBV KU981084
AKTGTQVQFEKIIAFMALITMIIDTERSDAIFKVLNKLKSVFQTMGDNVQTQ
>ISMV KT692938
AGTPSTRKLESAIAATALVMVIFDSNRCDVLIKILNKLKTIFAALNFGVRFE
>JYMV AB027007
AKRRGELELERVVAFIALVMMVFDSERSDCVVKILNKLKNIISSTDADVYHQ
>JVT KT222674
AKNAEQIKFERIVAFISLVLMAFDSERSDCVYKILMKLKNLIGTCEQDVHFQ
>JGMV Z26920
KGYAELKLEQVIAFATLLTMLYDADRSDAVYKILQKIKACTNICTQEVKHEG
>KjMV JF838187
SKTYLEKNFEKIIAFMALITMMFDDKKSDAVFKCLGKIKTVFGTMEDVVRLQ
>KoMV AB219545
RSANEAAFESIIAITSLVLMVFDQERSDCVYRILQKLRSLVGISGEIVRHQ
>LYSV AJ307057
AKGDFQHLEKMIALLVLLTMLFDANRSDAVYKILNKFKGVMSSIDKEPMLHQ
>LMoV AJ564636
AKPDGERNLERIMAIIALTMMVFDADRSDCVYKVLNKLKGLLNTVHQEPVKFQ
>LMV X97705
AKREDQANLERIIAFTALVMMMFDSERSDCVYRSLSKLKSLVSTCDDDVRHQ
>LuMV EU847625
SKVSAGEAKLEKTIAVAALIMMIFDSERSDYLYKTLNKLKSLVRTVDDDVYHQ
>LVY MF543013
HKGKSLVNLERIIALFTLALMIVDPDRSDGVYKLLTKLKSVITTATQEPMRFQ
>MeRV MF953305
AKSNEMIQLEKVIATVALIMMVFDQERSDCVYKILNKLKGVINTMNQDNFKFQ
>MWMV EF579955
AKTVNEKRMEQILAFVTLIMMFIDAEKSDCVYRVLNKFKGVVGTIEQDVYHQ
>NDV AM182028
YKKKNENDLEKIVATIALIMMIFDTDRSDAVFKILNKVKTVFSTFGERVQFQ
>NLSYV KC691259
VKRRSEQELEKVVAFIALILMMFDSERSDCVAKILQKLKNLISSAEPDVYHQ
>NYSV AM158908
TKRKSEQELERVVAFVALVLMMFDCERSDCVTKVLTKLKNLMSSVEPNVYHQ
>NoMV MN114634
ASRGNNAKYEQLIAFVALILMVFDADKSDCVYRILNKLKSLTGTLENSVKHE

>OYDV AJ510223
AKSKSETTLEQIVALCALMAMFFNTEKSDAVFKILSKIKNVFSSTDFPVQYQ
>OrMV JQ807997
VKNQQEQNLEKIIAFVTLLSMLYSPERSDSLFKILNKVKGVLGTIEGGVYHQ
>PanVY GQ916624
AKRNSEVRLEQAMAFVALILMIFDSEKSDCVYKVLSKLKNLMSIADTSVFHQ
>PcLMV DQ851494
SKNPFERNLEKVVAMMALFAMMFGSDKSSAVFNVLRNIKTVFGTLEDDVRYQ
>PLDMV KT633944
AKRKGEQNLEKIIAFVALVMMIFDSEKSDCVYKTLNKLRNLVATCDEPVAHQ
>PMNV MF509898
GKTAVQLQFEKIVAFMALLTMCIDAERSDAIFKILSKLKTVFTTMGEDVKVQ
>ParV1 MN549985
AKTDGERNLERVVAVSALLLMVFDAERSDVVYKVLNKLKNLIGVVEAEPMKFQ
>PVV MH286883
SKSATQVNFEKIIAFMALLTMCVDAERSDALFRILHKLKTVFGTMGEDVRIQ
>PRSV X67673
AKSDNEKKLEQVIAFITLILMMIDVDKSDCVYRILNKFKGVINSSNTNVYHQ
>PWV HQ122652
AKTQVQLQFEKIIAVLALITMCIDAERSDAIFRILSKLKMVFSTVGEDVKVQ
>PeMoV AF023848
AKTETELGLEKVVAYLALIAMIFDGERSDAVFRALSKLKTVFGTLGETVRYQ
>PepYMV AB541985
KSTSDIKSVEHVVAFISLVIMMFDAERSDCVFKTLNKFKGIISSLDSEVRHQ
>PMaV KT633868
SKGRGQNKLEQIIAMAALVMMMFDSERSDAVYRSLQKLKFLTTTADDCMTFQ
>PepMoV M96425
RSTEDLKKVEHIIAFVTLAIMLFDSERSDCVFKTLNKFKGVVCSLGSGVRHQ
>PTV AJ437280
DSKSDLKKIEHIIAFVSLAVMLFDVERSDCVFKSLNKFKGVISSLNSDVRHQ
>PlaMMV MH779625
AKSQEQAQLERIIAVIALILMVFDAERSDCVYKILNKLRTLVGISSSDTMKFQ
>PPV KP998124
SKRDSQANLERVVAFVALVMMLFDSERSDGVYKILNKLKGIMGSVDQAVHHQ
>PStV U34972
AKNANQLQFEKIVAFMALLTMVIDTERSDAIFKILSKLKTVFNTMGDNVQMQ
>PVA AJ296311
AKASEQVNFERIIAFISLVLMMFDRERSDCVYRSLTKLKSLMSTVENTVQFQ
>PVY X97895
RSTPGVKNLEQVVAFITLIIMMFDAERSDCVFKTLNKFKGIVSSMDHEVKHQ
>PYBV JX294310
SKSSEQVNFERIIAFVSLVLMMFDAERSDCVYRSLTKLKSLMGTVENTVHFQ
>AcVA MT513101
SKTKSELHLEKVVATMALFTMILDPEKSDAVFRVLSKVKTFFGTVEDTVRYQ
>AshMV MN853672
AKKSKGEATLERIVAFMSLIMMMFDADKSDYIYKVLNKLRSLTTTIETEELVRHQ
>PalMV MT790493
KKTVTALRFEQMIAFATLAIMLYDSERSDAVYKILQKIRACTGILSTEVEHQ
>PleMV LC573287
KKSYSEMRLEQVIAFATLITMLYDAERSDAVFKILQKIRSCTSIISREVEHQ
>SaLV KY562565
AKTEKQRQLEKIVAFMALLAMVIDSERSDAVSKVLSKLKSVFITMGEEVRVQ
>ScaMV AJ316084
SKRHEQQELERIIAFVALVLMMFDAERSDCVTKILNKVRNLVTTTESTVYHQ
>SYSV AJ865076
AKSQSEVTLEKIVAYVALFAMLFNSEKSDGVFKILSKLKTIFSTTDVHYQ
>SMV AJ507388
TKTAIQLQLEKIVAFMALITMCIDNERSEAVFKVLSKLKLVFSTMGEDVRAQ
>SCMoV GU181199
KSTPEVKNLEQVIAFITLITMLFDSERSDCVFKTLNKLKGIVSTLDCEVRHQ

>SuRBV KX856009
KSTAGVKNLEQIVAFVTLITMIFDSERSDCVFKTLNKLKGVVSTLDMEVRHQ
>SPFMV D86371
GKSRKEMQYERIIAFVSLLLMIVDSEKSDCVYKILQKLKGLMGTINNDVYHQ
>SPLV KC443039
AKSPTEAKYERIIAMIALFMMAFDAERSDCVYKILNKLRTLTNIAQQDVHHQ
>SPVC GU207957
GKSRKEIQYERIIAFISLLLMVIDSERSDCVYKILSKLRGLMGCIDGGVYHQ
>SPVG JQ824374
AKGLKEANYERIIAFIALILMVVDAERSDCVYKALNKLKGLMSTICGGPVYHQ
>SPV2 JN613807
AKSAKESSYERIIAFIALVLMVIDAERSDCVYKSLNKLKGLMGTIGDGVYHQ
>TLMV KM523548
KGKKEFAYMERIIAIITLLIMAFDAERSDGVYQILNKFKGVIGSAERETIRLE
>TEV KM282187
AKQPEIAYFEKIIAFITLVLMAFDAERSDGVFKILNKFKGILSSTEREIIYTQ
>TelMV DQ851493
GKTGTQVQFEKIIAFMALITMIIDLERSDALFRILSKLKTVFGTMGEDVRAQ
>TFMV AJ851866
AKTDGERNLERVVAVSALLLMIFDAERSDVVYKILNKLKTLIGVVETEPMKFQ
>TMosqV KT834407
RGTADIKKLEHIXAFISLVIMVFDADRSDCVFKTLNKFKGIISSMDSEVRHQ
>TVBMV EF219408
AVKRASEVRLEQIVAFIALVMMIFDNDRSDCVYRVLNKFKNIVGVADQEVIHQ
>TNSV JQ314463
KVTEGTKKIEQIVAFMTLVMMMFDAERSDCVFKTLNKLKSTISTMDYEVRHQ
>TuMV AF169561
AKRQSEQELERIIAFVALVLMMFDAERSDCVTKILNKLKGLVATVEPTVYHQ
>TVMV X04083
AKNTGQASLERIIAFVSLTLMLFDNERSDCVYKILTKFKGILGSVENNVRFQ
>UPV MK110656
GKTKVQMQLERIIAFMALITMFIDSERSDAVFNVLHKVKAVFGTLGEEVKVQ
>VDMV KF906523
VKQHSCQQLEKVVAFVALVMMMFDAERSDCVYRTLNKFKGIMGSIDSGVFHQ
>VVY EU564817
KNDQDLKKLEHIVAFITLVMMVFDSERSDCVFKTLNKFKGVVASLNSTVHHQ
>WMV AY437609
AKTATQLQLEKIVAFMALLTMCIDNERSDAVFKILSKLKTFFGTMGEEVKVQ
>WoSV LC159494
ARRKSEQDLEKIVAFVALILMMFDSERSDCVAKVLNKLKNIMSSADPTVYHQ
>WPMV AJ437279
DSKSDLKKIEHIIAFVSLAVMLFDVERSDCVFKSLNKFKGVIGSLNSDVRHQ
>WTMV DQ851495
ASKRPSEAKLEQIVAFIALLMMVFDGDRGDCVYKVLNKLRNVMGSVDNEAVNHQ
>WVMV AY656816
AKTKLQLQLEKIVAFMALLTMCIDNERSDAIFKILSKLKTIFGTMGEDVKAQ
>YMMV JX470965
AKRESEQRLEQIIAFIALVMMVFDNERSDCVYKVLNKLKNLMNTAEPVAHQ
>YMV U42596
SKRSEECKFEAIIAFIALVLMIFDAERSDCVYRSLTKLKSLVATTEVSHQ
>ZSV KU355553
AKTKNEQHLEKILAFITLVMMMVDPDKSDCVYKILNKFKGVVGTIEQDVYHQ
>ZTMV KC345607
AKTNNEKKLEQIIAFITLIMMMVDTDKSDCLYRILNKFKGIMASDATNAYHQ
>ZYMV AF127929
AKSAVQIQFEKIIAVLALLTMCFDAERSDAIFKILTKLKTVFGTVGETVRLQ
